# Inflammation mediates the effect of histidine on a lower risk of colorectal cancer

**DOI:** 10.3389/fnut.2025.1699087

**Published:** 2026-01-21

**Authors:** Chunlei He, Yudan Yang, Shuhui Chen, Bin Liu, Jing Guo, Ding Ye, Xiaohui Sun, Yingying Mao, Jiayu Li

**Affiliations:** 1 School of Public Health, Zhejiang Chinese Medical University, Hangzhou, China; 2 Wujing Community Healthcare Service Center of Minhang District, Shanghai, China

**Keywords:** cohort study, colorectal cancer, histidine, inflammation, mediation effect

## Abstract

**Background:**

Whether anti-inflammatory mechanisms mediate the protective effects of histidine against colorectal carcinogenesis remains unexplored. We aimed to assess the association between plasma histidine and colorectal cancer (CRC) and to evaluate the potential mediating role of inflammation.

**Methods:**

We conducted a prospective cohort study using data from the UK Biobank. A total of 261,082 cancer-free participants with plasma histidine data were included. Histidine concentrations were quantified using a nuclear magnetic resonance-based metabolic profiling platform. Incident CRC cases were identified through linkage to cancer registries. Cox proportional hazards regression models were used to assess the association between plasma histidine and CRC risk. Mediation analysis was performed using a regression-based approach with closed-form parameterization and delta method inference.

**Results:**

After a median follow-up of 13.3 years, 3,436 incident CRC cases were identified. An inverse association between plasma histidine and CRC risk was observed [Tertile 2 (T2) vs. T1: HR = 0.885; 95% CI = 0.816, 0.960; *P* = 0.003; T3 vs. T1: HR = 0.890; 95% CI = 0.820, 0.966; *P* = 0.005]. Mediation analysis identified neutrophils, leukocytes, and C-reactive protein (CRP) as significant mediators. CRP exhibited the largest mediation proportion (14.141%), followed by neutrophils (11.258%) and leukocytes (6.770%).

**Conclusions:**

Higher histidine levels are associated with a lower risk of CRC, and systemic inflammation mediates this association. These findings suggest that dietary interventions targeting histidine intake could offer a promising strategy for CRC prevention and burden reduction.

## 1 Background

Colorectal cancer (CRC) ranks as the third most prevalent malignancy and the second leading cause of cancer-related mortality globally (1). With an estimated 50% of CRC cases being preventable (2), there is substantial potential to reduce the risk of this common cancer by modifying key lifestyle factors, particularly diet (2), highlighting the critical role of nutritional interventions in CRC prevention.

Histidine, an essential amino acid obtained from dietary sources (such as meat, chicken, and fish) (3), exhibits multifaceted biological properties including antioxidant, anti-inflammatory, and glucoregulatory activities (4). Notably, two studies from the European Prospective Investigation into Cancer and Nutrition (EPIC) cohort revealed inverse associations between circulating histidine levels and CRC risk (5, 6). While these findings established an epidemiological link, the underlying biological mechanisms have not been fully elucidated and warrant further investigation.

Existing evidence highlights histidine's regulatory role in intestinal homeostasis. For instance, experimental studies have found that histidine suppressed NF-κB activation and mitigated macrophage-derived proinflammatory cytokine production, positioning it as a promising therapeutic agent for Crohn's disease (7). Conversely, histidine deficiency has been shown to inhibit intestinal antioxidant capacity and induce endoplasmic reticulum stress, inflammatory responses, apoptosis, and necroptosis (8). These findings collectively implicate inflammatory modulation as a pivotal mechanism linking histidine to gastrointestinal pathophysiology. Furthermore, substantial evidence has demonstrated that inflammatory biomarkers and signaling pathways promote tumor development (9). Key pro-inflammatory cytokines such as interleukin (IL)-6, IL-1β, and tumor necrosis factor-α (TNF-α) activate the nuclear factor-κB (NF-κB) and signal transducer and activator of transcription 3 (STAT3) (10). These activated transcription factors subsequently regulate multiple oncogenic processes including cancer cell survival, proliferation, angiogenesis, invasion, and metastasis (11, 12). Consequently, targeting inflammation-related molecules and pathways has emerged as a crucial strategy for cancer prevention and therapeutic intervention (9). However, whether such anti-inflammatory mechanisms mediate the protective effects of histidine against colorectal carcinogenesis remains to be elucidated, particularly at the population level.

Based on the preceding background, we hypothesized that histidine may exert a protective effect against CRC, potentially mediated through its anti-inflammatory properties. Specifically, we postulated that higher histidine levels reduce systemic inflammation, thereby lowering CRC risk. To test this hypothesis, we conducted a prospective cohort study within the UK Biobank (UKB) to: (1) examine the association between plasma histidine and CRC risk, and (2) assess the potential mediating roles of specific inflammatory biomarkers. This study aimed to provide population-level evidence on the biological mechanisms of histidine in CRC and to identify potential targets for CRC prevention.

## 2 Methods

### 2.1 Study participants

All UKB participants provided written informed consent, and the study protocol was approved by the North West Multi-Centre Research Ethics Committee. The biobank includes more than half a million middle-aged and older adults from the UK, with detailed questionnaires, physical measurements, and biological samples collected at baseline. Further details can be found at https://www.ukbiobank.ac.uk/.

This study initially included 502,355 participants. After excluding participants who withdrew from UKB (*n* = 224), those diagnosed with cancer (except non-melanoma skin cancer) prior to or at baseline (*n* = 23,797), individuals with missing plasma histidine data (*n* = 217,161), and histidine outliers (*n* = 91), a total of 261,082 participants remained for the primary analysis. Further exclusion of participants with missing inflammatory biomarkers data (*n* = 6,828–11,908) yielded a final analytical sample of 249,174–254,254 for the mediation analysis (Supplementary Figure S1).

### 2.2 Plasma histidine measurement

Plasma histidine was quantified using Nightingale Health's high-throughput nuclear magnetic resonance (NMR) platform in randomly selected EDTA plasma samples. Detailed methodology for the platform and the experimental procedures are described elsewhere (https://biobank.ndph.ox.ac.uk/ukb/label.cgi?id=220). Briefly, plasma samples stored at −80 °C were thawed at +4 °C overnight, then mixed and centrifuged to remove any precipitate. Samples were transferred into NMR tubes and mixed in a 1:1 ratio with a phosphate buffer with an automated liquid handler. The prepared samples were loaded into a temperature-controlled sample changer maintained at +6 °C. NMR spectra were acquired for each sample using a 500 MHz NMR spectrometer. Following automated quality control, histidine was quantified using Nightingale Health's proprietary software.

Histidine values outside four interquartile ranges (IQR) from the median were considered outliers and excluded from analyses. Prior to the association analyses, plasma histidine levels were adjusted for the NMR spectrometer by fitting a linear regression model with Ln(*x* + 1)-transformed concentrations as the dependent variable and spectrometer as the predictor. The scaled residuals derived from this model were then utilized as predictors (transformed histidine variable) in the subsequent analyses (13). This variable was analyzed both as a continuous measure and by tertile groups (T1–T3).

### 2.3 Colorectal cancer ascertainment

CRC incidence during follow-up was determined based on diagnostic codes [International Classification of Diseases, 10th Revision (ICD-10): C18-C20] from cancer registries (14). The follow-up time for each participant was defined as the period from study entry until the earliest occurrence of CRC diagnosis, the study's conclusion, loss to follow-up, or death.

### 2.4 Inflammatory biomarkers

Given that inflammation is a recognized pathway in colorectal carcinogenesis (15) and considering the availability of inflammatory biomarkers in the UKB, we selected the following inflammatory biomarkers as potential mediators of the histidine–CRC association: lymphocytes (10^9^ cells/L), monocytes (10^9^ cells/L), neutrophils (10^9^ cells/L), platelets (10^9^ cells/L), leukocytes (10^9^ cells/L), C-reactive protein (CRP) (mg/L). Detailed information on the instrumentation and methodology can be found in the UKB resources: https://biobank.ndph.ox.ac.uk/ukb/label.cgi?id=100081 and https://biobank.ndph.ox.ac.uk/ukb/refer.cgi?id=1227. Briefly, all samples were processed at the UKB central laboratory within 24 hours of blood collection. The Beckman Coulter LH750 hematology analyzers were employed for quantitative automated hematological analysis and leukocyte differential counting, enabling the measurement of lymphocytes, monocytes, neutrophils, platelets, and leukocytes. CRP was analyzed using the Beckman Coulter AU5800 platform with an immunoturbidimetric methodology. Due to the skewed distribution, CRP concentrations were Ln-transformed prior to analysis.

### 2.5 Covariates

Based on the previous studies (16, 17), we selected the following covariates: age, sex, UKB assessment center, ethnicity, body mass index (BMI), Townsend deprivation index, education level, alcohol drinking, cigarette smoking, physical activity, fruit intake, vegetable intake, processed meat intake, and unprocessed red meat intake. BMI (kg/m^2^) = Weight (kg)/Height^2^ (m^2^). Physical activity was defined as meeting any one of the following criteria: (1) at least 150 minutes of moderate activity per week; (2) at least 75 minutes of vigorous activity per week; or (3) an equivalent combination of (1) and (2) (18). The frequency categories for unprocessed and processed meats were recoded as follows: never = 0, less than once a week = 0.5, once a week = 1, 2–4 times a week = 3, 5–6 times a week = 5.5, and once or more daily = 7. The intake frequencies for beef, lamb/mutton, and pork were then summed to calculate the total frequency of unprocessed red meat consumption (19). Vegetable and fruit intake was converted into servings per day using the following equivalents: 1 serving of fresh fruit = 1 piece; 1 serving of dried fruit = 5 pieces; 1 serving of cooked or raw vegetables = 3 tablespoons (20).

### 2.6 Statistical analysis

To compare plasma histidine between the incident CRC cases and non-CRC participants, we employed the Wilcoxon rank-sum test. The association between plasma histidine and CRC risk was assessed using Cox proportional hazards models to estimate hazard ratios (HRs) with 95% confidence intervals (CIs). The proportional hazards assumption was evaluated with Schoenfeld residuals, and no violations were found. Model 1 was adjusted for age, sex, and UKB assessment center. Model 2 was additionally adjusted for ethnicity, BMI, Townsend deprivation index, education level, alcohol drinking, cigarette smoking, physical activity, fruit intake, vegetable intake, processed meat intake, and unprocessed red meat intake. We performed stratified analyses by age and sex and used the likelihood ratio test to assess potential interactions between plasma histidine and these factors.

Sensitivity analyses included: (1) Excluding incident CRC cases diagnosed during the first year of follow-up; (2) Additionally adjusting for fasting duration; (3) Additionally adjusting for cardiovascular diseases (ICD-10 codes: I20, I21, I22, I23, I24.1, I25, I46, I60, I61, I63, and I64) and type 2 diabetes (ICD-10 code: E11) (21); (4) Reanalyzing the data after excluding participants with missing covariates.

To evaluate whether inflammatory biomarkers mediate the association between histidine and CRC, we conducted a mediation analysis. Biomarkers that were significantly associated with both plasma histidine and CRC risk (*P* < 0.05 for each association) were considered candidate mediators. Using the *CMAverse* R package, we implemented a regression-based approach with closed-form parameterization and delta method inference to estimate the natural indirect effect (NIE) (risk ratio scale) and proportion mediated (22–24). Multiple imputation for missing covariates (via the *mice* package) and all other analyses were performed in R version 4.4.2, with a two-sided *P* < 0.05 defining statistical significance.

## 3 Results

Baseline characteristics of participants are presented in Supplementary Table S1. Individuals with higher plasma histidine levels exhibited distinct demographic and lifestyle traits. They were more likely to be male, be of White ethnicity, and have a college or university degree. They were also more likely to be current drinkers and physically active, but less likely to be current smokers. They tended to consume more processed and unprocessed red meats and fewer fruits. Following multiple imputation for missing covariates, the distribution patterns of these baseline characteristics are presented in Supplementary Table S2.

After a median follow-up of 13.3 years, 3,436 CRC incident cases were recorded. The distribution of plasma histidine levels between CRC cases and non-CRC participants is presented in Table 1. The CRC cases showed a median (IQR) of 0.064 (0.014), which was slightly but statistically significantly lower than that in the non-CRC participants [0.065 (0.014), *P* < 0.001].

**Table 1:** Distribution of plasma histidine levels in incident CRC cases and non-CRC participants.

**Histidine**	**Total participants**	**CRC cases**	**Non-CRC participants**	***P***
Minimum	0.021	0.035	0.021	
5th percentile	0.049	0.049	0.049	
10th percentile	0.053	0.052	0.053	
25th percentile	0.058	0.058	0.058	
75th percentile	0.072	0.072	0.072	
90th percentile	0.079	0.079	0.079	
95th percentile	0.083	0.083	0.083	
Maximum	0.120	0.112	0.120	
Median (IQR)	0.065 (0.014)	0.064 (0.014)	0.065 (0.014)	< 0.001

CRC, colorectal cancer; IQR, interquartile range; SD, standard deviation.

**Figure 1:**
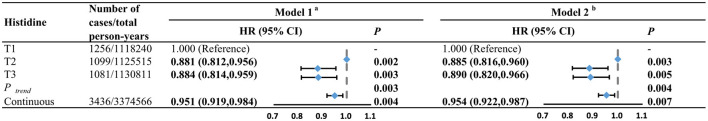
Association between plasma histidine and CRC risk. ^a^Model 1: Adjusted for age, sex, and UKB assessment center. ^b^Model 2: Adjusted for age, sex, UKB assessment center, ethnicity, Townsend deprivation index, education level, BMI, alcohol drinking, cigarette smoking, physical activity, fruit intake, vegetable intake, processed meat intake, and unprocessed red meat intake. BMI, body mass index; CI, confidence interval; CRC, colorectal cancer; HR, hazard ratio; UKB, UK Biobank.

The association between plasma histidine and CRC risk is presented in Figure 1. In the model adjusted for age, sex, and UKB assessment center, participants in the T2 and T3 of histidine exhibited a significantly lower risk of CRC compared to those in the T1 (T2: HR = 0.881; 95% CI = 0.812, 0.956; *P* = 0.002; T3: HR = 0.884; 95% CI = 0.814,0.959; *P* = 0.003). When analyzed as a continuous variable, histidine remained inversely associated with CRC risk (HR_Continuous_ = 0.951; 95% CI = 0.919, 0.984; *P* = 0.004). In the fully adjusted model, the inverse association between histidine and CRC risk was slightly attenuated but remained statistically significant (T2: HR = 0.885; 95% CI = 0.816, 0.960; *P* = 0.003; T3: HR = 0.890; 95% CI = 0.820, 0.966; *P* = 0.005). Similarly, when histidine was modeled as a continuous variable, the association persisted (HR_Continuous_ = 0.954; 95% CI = 0.922, 0.987; *P* = 0.007). The inverse association between plasma histidine and CRC risk remained robust across all sensitivity analyses (Supplementary Table S3).

Age-stratified analysis revealed a consistent association between plasma histidine and CRC risk in both participants aged < 60 years and those ≥60 years, with no significant interaction effect observed (*P*_interaction_ = 0.999) (Supplementary Table S4). In contrast, sex-stratified analysis showed a significant inverse association between plasma histidine and CRC risk exclusively in males (HR_Continuous_ = 0.908; 95% CI: 0.868, 0.950; *P* < 0.001), with a significant interaction between sex and histidine (*P*_interaction_ < 0.001) (Supplementary Table S5).

**Figure 2:**
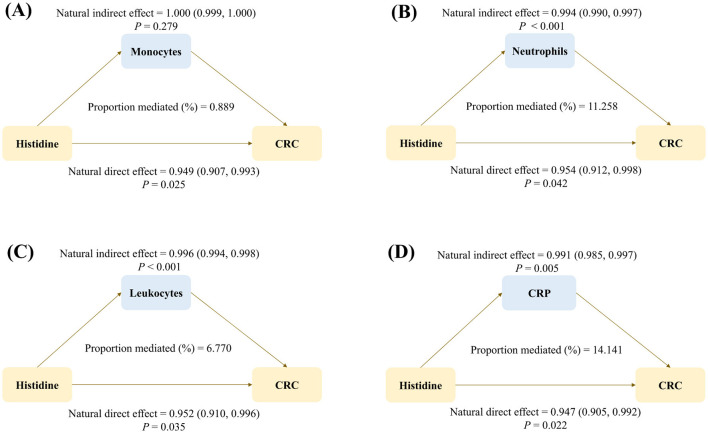
Mediating roles of inflammatory biomarkers [monocytes **(A)**, neutrophils **(B)**, leukocytes **(C)**, and CRP **(D)**] in the association between plasma histidine and CRC risk. All models were adjusted for age, sex, UKB assessment center, ethnicity, Townsend deprivation index, education level, BMI, alcohol drinking, cigarette smoking, physical activity, fruit intake, vegetable intake, processed meat intake, and unprocessed red meat intake. BMI, body mass index; CI, confidence interval; CRC, colorectal cancer; CRP, C-reactive protein; UKB, UK Biobank.

We also performed mediation analyses to assess whether inflammatory biomarkers mediate the association between plasma histidine and CRC risk. First, since inflammatory biomarker data were only available for subsets of participants (Supplementary Figure S1), we confirmed that the association between plasma histidine and CRC remained statistically significant in these subpopulations (Supplementary Table S6). Next, we examined the associations between plasma histidine and inflammatory biomarkers, as well as between inflammatory biomarkers and CRC risk. Four biomarkers—monocytes, neutrophils, leukocytes, and CRP—were significantly associated with both histidine and CRC risk (Table 2). All four biomarkers showed negative associations with histidine levels (all *P* < 0.001), and CRP showed the strongest association (β = −0.109, SE = 0.002, *P* < 0.001). In addition, all four biomarkers were positively associated with CRC risk (all *P* < 0.05), with monocytes showing the highest HR (HR = 1.150, 95% CI: 1.002, 1.320, *P* = 0.047). We then estimated the mediating effects of these four inflammatory biomarkers on the histidine–CRC relationship, as summarized in Figure 2. For neutrophils, the NIE was 0.994 (95% CI = 0.990, 0.997) and the natural direct effect (NDE) of histidine on CRC was 0.954 (95% CI = 0.912, 0.998). For leukocytes, the NIE was 0.996 (95% CI = 0.994, 0.998) and the NDE was 0.952 (95% CI = 0.910, 0.996). For CRP, the NIE was 0.991 (95% CI = 0.985, 0.997) and the NDE was 0.947 (95% CI = 0.905, 0.992). Overall, CRP exhibited the highest proportion mediated (14.141%), followed by neutrophils (11.258%) and leukocytes (6.770%).

**Table 2:** Associations between plasma histidine and inflammatory biomarkers and between inflammatory biomarkers and CRC risk.

**Inflammatory biomarkers**	**Associations between plasma histidine and inflammatory biomarkers**		**Associations between inflammatory biomarkers and CRC risk**	
	β **(SE) of histidine**^b^	***P***	**HR (95% CI) of inflammatory biomarker** ^c^	***P***
Lymphocytes	−0.001 (0.002)	0.455	1.002 (0.964, 1.041)	0.910
Monocytes	−0.004 (0.0004)	< 0.001	1.150 (1.002, 1.320)	0.047
Neutrophils	−0.091 (0.003)	< 0.001	1.051 (1.027, 1.077)	< 0.001
Platelets	−1.406 (0.115)	< 0.001	1.000 (1.000, 1.001)	0.348
Leukocytes	−0.097 (0.004)	< 0.001	1.027 (1.012, 1.042)	< 0.001
CRP^a^	−0.109 (0.002)	< 0.001	1.052 (1.014, 1.091)	0.007

BMI, body mass index; CI, confidence interval; CRC, colorectal cancer; CRP, C-reactive protein; HR, hazard ratio; SE, standard error; UKB, UK Biobank.

^a^Ln-transformed CRP was used to analyze its associations with plasma histidine and CRC risk.

^b^Adjusted for age, sex, UKB assessment center, ethnicity, Townsend deprivation index, education level, BMI, alcohol drinking, cigarette smoking, physical activity, fruit intake, vegetable intake, processed meat intake, and unprocessed red meat intake.

^c^Adjusted for age, sex, UKB assessment center, ethnicity, Townsend deprivation index, education level, BMI, alcohol drinking, cigarette smoking, physical activity, fruit intake, vegetable intake, processed meat intake, unprocessed red meat intake, and plasma histidine.

## 4 Discussion

Our cohort study found that plasma histidine levels were inversely associated with the risk of CRC. A significant interaction between sex and histidine was observed for CRC risk. Furthermore, we identified specific inflammatory biomarkers—CRP, neutrophils, and leukocytes—as mediators of this relationship.

Our study confirms the previously reported inverse association between circulating histidine and CRC risk, consistent with findings from the EPIC cohort and UKB validation analyses (5, 6). While reinforcing this evidence, our research extends prior work through key methodological refinements: (1) We included a larger number of participants (261,082 vs. 111,323) by applying minimal exclusion criteria, thereby improving the representativeness of our sample. (2) We implemented a longer follow-up period (mean of 12.9 vs. 9.7 years). This longer follow-up resulted in a greater number of documented cancer cases, which enhanced the statistical power of our study and reduced the likelihood of reverse causality. (3) We comprehensively adjusted for dietary confounders that were not adequately addressed in previous analyses. Beyond its role in CRC onset, histidine depletion also appears to be involved in CRC progression. Multiple studies have consistently shown declining circulating histidine levels in advanced-stage CRC (25–27). Additionally, tissue metabolomics analysis has identified histidine as a discriminant metabolite for differentiating between CRC stages (28).

Our study provides novel mechanistic insights by demonstrating that histidine influences CRC development through modulation of systemic inflammation, with CRP, neutrophils, and leukocytes identified as key mediating biomarkers. This finding is supported by a prognostic study of 336 CRC patients, which showed that lower serum histidine levels were correlated with elevated systemic inflammatory markers, such as CRP, IL-6, IL-8, and the neutrophil-to-lymphocyte ratio (27). Animal studies further support the important role of histidine in the relationships between inflammation and gastrointestinal diseases. For instance, a deficiency in dietary histidine was found to significantly lower intestinal levels of the anti-inflammatory factors TGF-β and IL-10 while simultaneously increasing the pro-inflammatory cytokine TNF-α (8). Conversely, in a colitis model, histidine suppressed LPS-induced production of TNF-α and IL-6 in macrophages and inhibited LPS-induced NF-κB activation in these cells (7). On the other hand, emerging evidence highlights histidine's crucial role in maintaining intestinal epithelial homeostasis. An *in vitro* study using the rat intestinal epithelial cell-6 (IEC-6) revealed that histidine deprivation impaired epithelial restitution through downregulation of TGF-β1, a growth factor essential for mucosal repair (29). Furthermore, an animal study demonstrated that enteral nutrition-mediated accumulation of *F. rodentium* exerted colitis-protective effects via gut microbiota-dependent histidine biosynthesis (30). This collective evidence establishes the biological plausibility linking histidine to colorectal carcinogenesis, particularly through inflammatory mechanisms. However, epidemiological validation of this hypothesis remains limited, highlighting the need for large-scale prospective studies to elucidate the causal relationship between histidine-regulated inflammation and CRC.

Notably, a recent integrated metagenomic and metabolomic analysis revealed the enrichment of imidazole propionate (ImP) in both colorectal adenoma and CRC (31). ImP, a gut microbiota-derived histidine metabolite, has garnered significant research attention in recent years. Population studies have linked elevated ImP levels to various metabolic disorders, including elevated diastolic blood pressure (32), type 2 diabetes (33), and heart failure (34), highlighting its potential as a circulating biomarker of gut dysbiosis. Mechanistic studies further associate ImP with insulin resistance, impaired glucose metabolism, chronic inflammation, and intestinal barrier dysfunction (35). Notably, the latter two mechanisms—chronic inflammation and intestinal barrier impairment—may help further elucidate the observed association between histidine and CRC. However, the limitations of the NMR-based platform prevented the measurement of ImP levels in the UKB—a critical gap that warrants future research.

Sex hormones may modulate the role of inflammation in carcinogenic pathways and regulate the gut microbiota, thereby influencing the “histidine—inflammation—CRC” axis. This interaction could explain the sex-specific differences in the protective effect of histidine against CRC observed in our stratified analysis. For instance, prior studies indicate that estrogen mitigates intestinal inflammation, enhances gut microbiota diversity and the abundance of beneficial commensal bacteria, and reduces pathogen enrichment. In contrast, androgen has been shown to disrupt intestinal microecology, exacerbate colonic inflammation, and promote tumor growth (36).

Several limitations should be acknowledged in this study. First, the UKB lacks dietary intake data on histidine, preventing a comprehensive assessment of its impact on CRC from an external exposure perspective. Second, although NMR-based metabolomic profiling offers high-throughput advantages, it may introduce measurement errors. Third, the UKB also lacks data on histidine metabolic derivatives, which hinders a thorough evaluation of the entire histidine metabolic pathway in relation to CRC risk. Fourth, despite our rigorous adjustment for known covariates, we cannot rule out the possibility of residual confounding. Fifth, the observational nature of the cohort design means reverse causality cannot be excluded. Sixth, our findings are based on the UKB, which may be subject to the health volunteer effect, potentially limiting the generalizability of our results. Finally, the UKB cohort is predominantly of European/Caucasian ancestry, which may limit the extrapolation of our findings to other populations.

## 5 Conclusions

Higher plasma histidine levels are associated with a reduced risk of CRC, with systemic inflammation serving as a significant mediator. These findings suggest that incorporating histidine-rich foods into daily diets could serve as a practical strategy to modulate inflammation and subsequently reduce CRC risk.

## Data Availability

The original contributions presented in the study are included in the article/Supplementary material, further inquiries can be directed to the corresponding author/s.
