# Supplementary material for: Inflammation mediates the effect of histidine on a lower risk of colorectal cancer
Source: Front Nutr. 2026 Jan 21;12:1699087. doi: 10.3389/fnut.2025.1699087 (PMC12867850; doi:10.3389/fnut.2025.1699087)
Supplement: Supplementary file 1 [file Table_1.docx]

**Title: Inflammation Mediates the Association Between Histidine and Colorectal Cancer**

| **Content** | **Page** |
| --- | --- |
| Table S1. Baseline characteristics of participants in the present study (N =261082) | 2-3 |
| Table S2. Baseline characteristics of participants after multiple imputation for missing covariates (N =261082) | 4-5 |
| Table S3. Sensitivity analysis of the association between plasma histidine and CRC risk | 6-7 |
| Table S4. Stratified analysis of the association between plasma histidine and CRC risk by age | 8 |
| Table S5. Stratified analysis of the association between plasma histidine and CRC risk by sex | 9 |
| Table S6. Association between plasma histidine and CRC risk in subpopulations with available data on inflammatory biomarkers | 10 |
| Figure S1. Flowchart of inclusion and exclusion of study participants | 11 |

| **Table S1. Baseline characteristics of participants in the present study (N =261082)** | | | |
| --- | --- | --- | --- |
| **Characteristics** | **Tertiles of plasma histidine** | | |
|  | **T1** | **T2** | **T3** |
| N | 87029 | 87026 | 87027 |
| Age (years), median (IQR) | 58 (13) | 58 (13) | 57 (13) |
| Sex, n (%) |  |  |  |
| Female | 51697 (59.4) | 46739 (53.7) | 41547 (47.7) |
| Male | 35332 (40.6) | 40287 (46.3) | 45480 (52.3) |
| Ethnicity, n (%) |  |  |  |
| White | 82058 (94.3) | 82322 (94.6) | 82476 (94.8) |
| Others | 4584 (5.3) | 4302 (4.9) | 4161 (4.8) |
| Missing | 387 (0.4) | 402 (0.5) | 390 (0.4) |
| Townsend deprivation index, median (IQR) ^a^ | -2.1 (4.1) | -2.2 (4.1) | -2.2 (4.1) |
| Education level, n (%) |  |  |  |
| College or university degree | 26368 (30.3) | 27719 (31.9) | 28841 (33.1) |
| Others | 59596 (68.5) | 58283 (67.0) | 57248 (65.8) |
| Missing | 1065 (1.2) | 1024 (1.2) | 938 (1.1) |
| BMI (kg/m^2^), median (IQR) ^b^ | 26.8 (6.2) | 26.7 (5.7) | 26.8 (5.4) |
| Alcohol drinking, n (%) |  |  |  |
| Never | 4122 (4.7) | 3813 (4.4) | 3361 (3.9) |
| Previous | 3294 (3.8) | 3003 (3.5) | 2920 (3.4) |
| Current | 79379 (91.2) | 80003 (91.9) | 80568 (92.6) |
| Missing | 234 (0.3) | 207 (0.2) | 178 (0.2) |
| Cigarette smoking, n (%) |  |  |  |
| Never | 47015 (54.0) | 47539 (54.6) | 47866 (55.0) |
| Previous | 29776 (34.2) | 29949 (34.4) | 30001 (34.5) |
| Current | 9792 (11.3) | 9109 (10.5) | 8772 (10.1) |
| Missing | 446 (0.5) | 429 (0.5) | 388 (0.4) |
| Physical activity, n (%) |  |  |  |
| No | 30665 (35.2) | 30548 (35.1) | 30504 (35.1) |
| Yes | 46012 (52.9) | 47043 (54.1) | 47813 (54.9) |
| Missing | 10352 (11.9) | 9435 (10.8) | 8710 (10.0) |
| Fruit intake, n (%) |  |  |  |
| < 4 servings/day | 70693 (81.2) | 71048 (81.6) | 71441 (82.1) |
| ≥ 4 servings/day | 15122 (17.4) | 14895 (17.1) | 14533 (16.7) |
| Missing | 1214 (1.4) | 1083 (1.2) | 1053 (1.2) |
| Vegetable intake, n (%) |  |  |  |
| < 4 servings/day | 82951 (95.3) | 83121 (95.5) | 83035 (95.4) |
| ≥ 4 servings/day | 2790 (3.2) | 2721 (3.1) | 2782 (3.2) |
| Missing | 1288 (1.5) | 1184 (1.4) | 1210 (1.4) |
| Processed meat intake, n (%) |  |  |  |
| > 1 times/week | 26126 (30.0) | 27130 (31.2) | 28723 (33.0) |
| ≤ 1 time/week | 60576 (69.6) | 59582 (68.5) | 58038 (66.7) |
| Missing | 327 (0.4) | 314 (0.4) | 266 (0.3) |
| Unprocessed red meat intake, n (%) |  |  |  |
| > 1.5 times/week | 43595 (50.1) | 43814 (50.3) | 44247 (50.8) |
| ≤ 1.5 times/week | 42480 (48.8) | 42333 (48.6) | 42023 (48.3) |
| Missing | 954 (1.1) | 879 (1.0) | 757 (0.9) |
| *Abbreviation:* BMI, body mass index; IQR, interquartile range; T1, the first tertile; T2, the second tertile; T3, the third tertile.  ^a^ There were 320 missing values for the Townsend deprivation index.  ^b^ There were 994 missing values for the BMI. | | | |

| **Table S2. Baseline characteristics of participants after multiple imputation for missing covariates (N =261082)** | | | |
| --- | --- | --- | --- |
| **Characteristics** | **Tertiles of plasma histidine** | | |
|  | **T1** | **T2** | **T3** |
| N | 87029 | 87026 | 87027 |
| Ethnicity, n (%) |  |  |  |
| White | 82418 (94.7) | 82695 (95.0) | 82851 (95.2) |
| Others | 4611 (5.3) | 4331 (5.0) | 4176 (4.8) |
| Townsend deprivation index, median (IQR) | -2.1 (4.1) | -2.2 (4.1) | -2.2 (4.1) |
| Education level, n (%) |  |  |  |
| College or university degree | 26737 (30.7) | 28045 (32.2) | 29169 (33.1) |
| Others | 60292 (69.3) | 58981 (67.8) | 57858 (66.9) |
| BMI (kg/m^2^), median (IQR) | 26.8 (6.2) | 26.7 (5.7) | 26.8 (5.4) |
| Alcohol drinking, n (%) |  |  |  |
| Never | 4133 (4.7) | 3822 (4.4) | 3369 (3.9) |
| Previous | 3304 (3.8) | 3010 (3.5) | 2922 (3.4) |
| Current | 79592 (91.5) | 80194 (92.1) | 80736 (92.8) |
| Cigarette smoking, n (%) |  |  |  |
| Never | 47248 (54.3) | 47776 (54.9) | 48081 (55.2) |
| Previous | 29929 (34.4) | 30101 (34.6) | 30125 (34.6) |
| Current | 9852 (11.3) | 9149 (10.5) | 8821 (10.1) |
| Physical activity, n (%) |  |  |  |
| No | 34793 (40.0) | 34267 (39.4) | 33954 (39.0) |
| Yes | 52236 (60.0) | 52759 (60.6) | 53073 (61.0) |
| Fruit intake, n (%) |  |  |  |
| < 4 servings/day | 71682 (82.4) | 71966 (82.7) | 72291 (83.1) |
| ≥ 4 servings/day | 15347 (17.6) | 15060 (17.3) | 14736 (16.9) |
| Vegetable intake, n (%) |  |  |  |
| < 4 servings/day | 84183 (96.7) | 84253 (96.8) | 84196 (96.7) |
| ≥ 4 servings/day | 2846 (3.3) | 2773 (3.2) | 2831 (3.3) |
| Processed meat intake, n (%) |  |  |  |
| > 1 times/week | 26233 (30.1) | 27229 (31.3) | 28797 (33.1) |
| ≤ 1 time/week | 60796 (69.9) | 59797 (68.7) | 58230 (66.9) |
| Unprocessed red meat intake, n (%) |  |  |  |
| > 1.5 times/week | 44089 (50.7) | 44251 (50.8) | 44627 (51.3) |
| ≤ 1.5 times/week | 42940 (49.3) | 42775 (49.2) | 42400 (48.7) |
| *Abbreviation:* BMI, body mass index; IQR, interquartile range; T1, the first tertile; T2, the second tertile; T3, the third tertile. | | | |

| **Table S3.** Sensitivity analysis of the association between plasma histidine and CRC risk | | | |
| --- | --- | --- | --- |
|  | **Number of cases/total person-years** | **HR (95% CI)** | ***P*** |
| ***Excluding incident CRC cases during the first year of follow-up ^a^*** | | | |
| T1 | 1166/1118443 | 1.000 (Reference) | - |
| T2 | 1032/1125420 | **0.896 (0.823,0.974)** | **0.010** |
| T3 | 1023/1130592 | **0.906 (0.833,0.986)** | **0.023** |
| *P _trend_* |  |  | **0.020** |
| Continuous | 3221/3374455 | **0.962 (0.929,0.997)** | **0.031** |
| ***Additionally adjusting for fasting duration ^b^*** | | | |
| T1 | 1256/1118240 | 1.000 (Reference) | - |
| T2 | 1099/1125515 | **0.886 (0.817,0.961)** | **0.003** |
| T3 | 1081/1130811 | **0.891 (0.821,0.968)** | **0.006** |
| *P _trend_* |  |  | **0.005** |
| Continuous | 3436/3374566 | **0.955 (0.923,0.988)** | **0.008** |
| ***Additionally adjusting for cardiovascular diseases and type 2 diabetes ^c^*** | | | |
| T1 | 1256/1118240 | 1.000 (Reference) | - |
| T2 | 1099/1125515 | **0.885 (0.816,0.960)** | **0.003** |
| T3 | 1081/1130811 | **0.889 (0.819,0.965)** | **0.005** |
| *P _trend_* |  |  | **0.004** |
| Continuous | 3436/3374566 | **0.954 (0.922,0.987)** | **0.007** |
| ***Reanalyzing the data after excluding participants with missing covariates ^a^*** | | | |
| T1 | 1081/962918 | 1.000 (Reference) | - |
| T2 | 928/969190 | **0.868 (0.795,0.948)** | **0.002** |
| T3 | 927/972787 | **0.887 (0.812,0.969)** | **0.008** |
| *P _trend_* |  |  | **0.006** |
| Continuous | 2936/2904895 | **0.950 (0.915,0.986)** | **0.006** |
| *Abbreviation:* BMI, body mass index; CRC, colorectal cancer; CI, confidence interval; HR, hazard ratio; T1, the first tertile; T2, the second tertile; T3, the third tertile; UKB, UK biobank. | | | |
| ^a^ Adjusted for age, sex, UKB assessment center, ethnicity, Townsend deprivation index, education level, BMI, alcohol drinking, cigarette smoking, physical activity, fruit intake, vegetable intake, processed meat intake, and unprocessed red meat intake. | | | |
| ^b^ Adjusted for age, sex, UKB center, ethnicity, Townsend deprivation index, education level, BMI, alcohol drinking, cigarette smoking, physical activity, fruit intake, vegetable intake, processed meat intake, unprocessed red meat intake, and fasting duration. | | | |
| ^c^ Adjusted for age, sex, UKB center, ethnicity, Townsend deprivation index, education level, BMI, alcohol drinking, cigarette smoking, physical activity, fruit intake, vegetable intake, processed meat intake, unprocessed red meat intake, cardiovascular diseases, and type 2 diabetes. | | | |

| **Table S4.** Stratified analysis of the association between plasma histidine and CRC risk by age | | | | | | | | |
| --- | --- | --- | --- | --- | --- | --- | --- | --- |
| **Histidine** | **< 60 years** | | |  | **≥ 60 years** | | | ***P*** ***_interaction_*** |
|  | **Number of cases/total person-years** | **HR (95% CI) ^a^** | ***P*** |  | **Number of cases/total person-years** | **HR (95% CI) ^a^** | ***P*** |  |
| T1 | 414/616858 | 1.000 (Reference) | - |  | 842/501382 | 1.000 (Reference) | - | - |
| T2 | 388/655329 | **0.869 (0.756,0.998)** | **0.047** |  | 711/470186 | **0.876 (0.793,0.969)** | **0.010** | - |
| T3 | 418/699288 | **0.858 (0.748,0.984)** | **0.029** |  | 663/431523 | **0.869 (0.785,0.963)** | **0.007** | - |
| *P _trend_* |  |  | **0.030** |  |  |  | **0.006** |  |
| Continuous | 1220/1971475 | **0.942 (0.890,0.996)** | **0.037** |  | 2216/1403091 | **0.943 (0.903,0.984)** | **0.007** | 0.999 |
| *Abbreviation:* BMI, body mass index; CRC, colorectal cancer; CI, confidence interval; HR, hazard ratio; T1, the first tertile; T2, the second tertile; T3, the third tertile; UKB, UK biobank.  ^a^ Adjusted for sex, UKB assessment center, ethnicity, Townsend deprivation index, education level, BMI, alcohol drinking, cigarette smoking, physical activity, fruit intake, vegetable intake, processed meat intake, and unprocessed red meat intake. | | | | | | | | |

| **Table S5.** Stratified analysis of the association between plasma histidine and CRC risk by sex | | | | | | | | |
| --- | --- | --- | --- | --- | --- | --- | --- | --- |
| **Histidine** | **Females** | | |  | **Males** | | | ***P*** ***_interaction_*** |
|  | **Number of cases/total person-years** | **HR (95% CI) ^a^** | ***P*** |  | **Number of cases/total person-years** | **HR (95% CI) ^a^** | ***P*** |  |
| T1 | 571/671865 | 1.000 (Reference) | - |  | 685/446375 | 1.000 (Reference) | - | - |
| T2 | 462/609869 | 0.923 (0.816,1.043) | 0.199 |  | 637/515646 | **0.853 (0.766,0.951)** | **0.004** | - |
| T3 | 432/544525 | 1.007 (0.888,1.141) | 0.916 |  | 649/586286 | **0.815 (0.732,0.908)** | **< 0.001** | - |
| *P _trend_* |  |  | 0.987 |  |  |  | **< 0.001** |  |
| Continuous | 1465/1826259 | 1.022 (0.970,1.076) | 0.419 |  | 1971/1548307 | **0.908 (0.868,0.950)** | **< 0.001** | **< 0.001** |
| *Abbreviation:* BMI, body mass index; CRC, colorectal cancer; CI, confidence interval; HR, hazard ratio; T1, the first tertile; T2, the second tertile; T3, the third tertile; UKB, UK biobank. | | | | | | | | |
| ^a^ Adjusted for age, UKB assessment center, ethnicity, Townsend deprivation index, education level, BMI, alcohol drinking, cigarette smoking, physical activity, fruit intake, vegetable intake, processed meat intake, and unprocessed red meat intake. | | | | | | | | |

| **Table S6.** Association between plasma histidine and CRC risk in subpopulations with available data on inflammatory biomarkers | | | |
| --- | --- | --- | --- |
| **Inflammatory biomarkers** | **Number of cases/total person-years** | **HR _Continuous_ (95% CI) ^a^** | ***P*** |
| Lymphocytes | 3336/3282955 | **0.960 (0.927,0.993)** | **0.019** |
| Monocytes | 3336/3282955 | **0.960 (0.927,0.993)** | **0.019** |
| Neutrophils | 3336/3282955 | **0.960 (0.927,0.993)** | **0.019** |
| Platelets | 3340/3286970 | **0.961 (0.928,0.994)** | **0.023** |
| Leukocytes | 3340/3286969 | **0.961 (0.928,0.994)** | **0.023** |
| CRP | 3274/3224066 | **0.954 (0.921,0.988)** | **0.008** |
| *Abbreviation:* BMI, body mass index; CI, confidence interval; CRC, colorectal cancer; CRP, C-reactive protein; HR, hazard ratio; UKB, UK biobank. | | | |
| ^a^ Adjusted for age, sex, UKB assessment center, ethnicity, Townsend deprivation index, education level, BMI, alcohol drinking, cigarette smoking, physical activity, fruit intake, vegetable intake, processed meat intake, and unprocessed red meat intake. | | | |

**
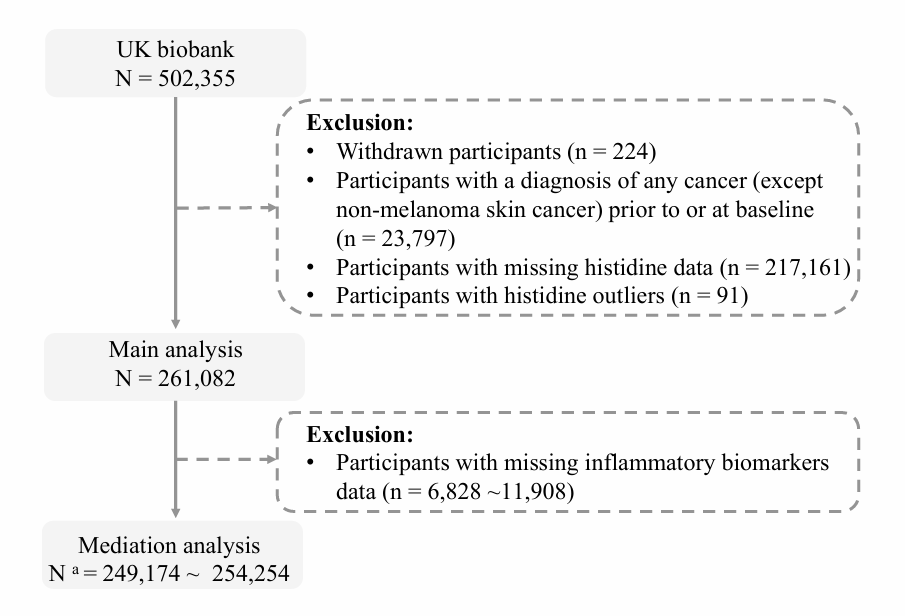
**

**Figure S1.** Flowchart of inclusion and exclusion of study participants

^a^ Due to varying degrees of missing data across inflammatory biomarkers, the final sample size included in the mediation analysis differed for each marker. The subpopulation sizes were as follows: Lymphocytes, 253,924; Monocytes, 253,924; Neutrophils, 253,924; Platelets, 254,254; Leukocytes, 254,254; CRP, 249,174.
